# Vertical wind profile distribution within canopy layer based on representative geometry model using wind tunnel experiment

**DOI:** 10.1038/s41598-024-83400-9

**Published:** 2025-01-07

**Authors:** S. A. Zaki, Y. M. H’ng, A. F. Mohammad, Jorge Alfredo Ardila‑Rey, Noor Alam, Mardiana Idayu Ahmad

**Affiliations:** 1 Department of Mechanical Precision Engineering, Malaysia-Japan International Institute of Technology, Universiti Teknologi Malaysia, Jalan Sultan Yahya Petra , 54100 Kuala Lumpur, Malaysia; 2 Department of Electrical Engineering, Universidad Técnica Federico Santa María, 8940000 Santiago de Chile, Chile; 3 Environmental Technology Division, School of Industrial Technology, Universiti Sains Malaysia, 11800 Penang, Malaysia

**Keywords:** Wind tunnel experiment, Wind profile distribution, Tropical climate, Canopy layer, Realistic geometry model, Mechanical engineering, Atmospheric dynamics, Natural hazards, Fluid dynamics

## Abstract

This study examined the mean and turbulent wind speed distribution within the canopy height of a tropical urban campus based on a representative geometry model via wind tunnel experiments. The vertical wind profiles were analysed around two high-rise buildings, Menara Razak (MR) and Residensi Tower (RT) at both wind directions (22.5° and 202.5°). To examine the influence of high-rise buildings on strong wind, the collected data of mean wind speed (*u*), root mean square (*u*_*rms*_), and skewness (*SK*) were analysed. Effects of the wind direction, building layout or arrangement and building geometry under the canopy height were also examined. The results show that the building layout influenced the wind distribution within the target site, and the approaching wind flow direction also influenced the wind flow interaction with the building. The height of the target building (*H*) influenced the distance traveled by the vortices in the wake flow. For the MR and RT cases, the vortices could be affected up to a minimum distance of *H* and 1.5*H*, respectively. The study demonstrates that the building layout significantly influences the distribution of wind speeds within the canopy height of a tropical urban campus.

## Introduction

The complexity of urban morphologies influences the microclimate within the canopy, leading towards changes in wind speeds, urban air quality, and the comfortability of the living space [1–5]. To ensure a sustainable quality of life for the communities, it is essential to design an urban space that regulates the optimum wind conditions. Some related pedestrian wind case study frameworks with standard practices and criteria have been thoroughly reviewed [6–8]. For example, Janssen et al. [6] analyzed the existing wind comfort criteria with the case study of Eindhoven University of Technology campus with computational fluid dynamics (CFD). They found discrepancies in assessing pedestrian wind comfort among the selected criteria, which led to inaccuracies or differing conclusions. Blocken et al. [7] designed a simulation framework that integrates best practice guidelines for conducting reliable CFD studies on identifying wind comfort levels. The framework includes validating CFD results with long-term on-site wind speed measurements. Generally, the standardization in wind comfort assessment methodologies is needed to achieve more reliable urban design outcomes.

One of the key contributors to local wind discomfort or disturbance within an urban area is the intensity of high-rise buildings due to the urban development plans to cater for increasing population [9–12]. The strong winds that are significantly higher (> 23 m/s) than normal (> 10 m/s) wind speed induced by high-rise buildings tend to deflect towards the ground level and its surroundings [1, 13, 14], posing risks not only to the public property losses but also endangering the safety of pedestrians. For instance, Kuo et al. [13] demonstrated that one of the main factors affecting wind speeds experienced by pedestrians is spacing between buildings for which they established a threshold for minimizing strong wind regions downstream of high-rise structures. The interaction between wind flow and urban structures or surfaces is often associated with different scales of turbulence and eddies that govern a large scale of wind flow [15] and fluctuate in speed and direction, causing wind gustiness. Within an urban canopy, there are two related turbulence scales: mesoscale turbulence, which refers to wakes or eddies due to large man-made structures such as buildings, and microscale turbulence, which is induced by surface roughness, vegetation, or smaller objects.

The often overlooked impact of wind and its characteristics within the urban canopy height may be a significant concern, as severe wind events might lead to unfavourable accidents or losses (human safety or damage of infrastructure) [16–18]. Yang et al. [17] explored potential severe wind events due to isolated high-rise buildings through the development of generic models aimed at predicting pedestrian-level wind. The proposed models provided effective estimates for the speed-up area (i.e., a zone where wind speeds are significantly higher) and maximum wind speed ratio relevant to pedestrian safety. Nevertheless, the wind distribution pattern during strong wind events varies due to the complex urban morphological features such as building geometry and arrangement. Multiple buildings can interfere with wind flow, creating low wind speed areas, particularly in street canyons where buildings are aligned in a way that obstructs airflow. For instance, the interaction of multiple buildings with wind flow creates a wake flow. Zaki et al. [19] found that a wake flow known as skimming flow exists in random building arrays with multiple building heights. By examining the drag coefficient of several random building arrays at varying densities via wind tunnel experiment, they showed that the drag coefficient decreases after a peak value and with a further increase in building density; this phenomenon is attributed to the skimming flow. This can potentially affect the pedestrian-level wind speed. Due to urban complexity, Yuan et al. [20] proposed a new modelling-mapping approach that can estimate the pedestrian-level wind speed within an urban area; this was adopted in Hong Kong case studies where the effect of a new building on the wind distribution can be evaluated.

Experts have discussed certain literature compilations regarding the flow structure characteristics around individual buildings [17, 21, 22]. Urban wind effects vary depending on the building geometry and morphology [23–29]. Comprehensive reviews and comparisons of the techniques and applications that could be employed in such studies were presented by [1] and [30]. The purpose of each urban wind environment study may vary differently according to the study’s interests. Table 1 summaries the wind studies in the past decade.

**Table 1:** Summary of the previous studies focusing on flow structure characteristics around buildings.

Author(s)/year	Interest of studies			Methods			Simplified model	Real model
	Wind comfort	Ventilation	Unfavourable high wind	CFD	WTE	Field Measurement		
Fadl and Karadelis [46]	✓	–	–	✓	–	–	Target building	–
Janssen et al. [47]	–	–	✓	✓	–	✓	–	Tower
Abd Razak et al. [68]	–	✓	–	✓	–	–	Block arrays	–
Kuo et al. [69]		–	✓	–	✓	–	Target building	–
Iqbal and Chan [70]	✓	–	–	✓	✓	–	Target building	–
Yuan et al. [20]	–	✓	–	✓	–	–	–	Districts
Ahmad et al. [49]	–		✓	✓	–	–	–	Township
Ikegaya et al. [71]	–	✓	–	✓	–	–	Block arrays	–
Ikegaya et al. [43]	–	–	✓	✓	–	–	Block arrays	–
Mittal et al. [62]	✓	–	–	✓	✓	–	Isolated block	–
Tamura et al. [23]	–	–	✓	–	✓	–	Single building	–
Ikegaya et al. [21]	–	–	✓	✓		–	Isolated block	–
Kuo et al. [13]	–	✓	–	–	✓	–	Building arrays	–
Salim et al. [72]	–	✓	–	✓		–	Block arrays	–
Chen and Mak [73]	✓		–	✓	✓	–	Target buildings	–
Ghalam et al. [29]	–	✓	–	✓		–	Block arrays	–
Tominaga and Shirzadi [12]	–	–	✓	–	✓	–	Block arrays	–
Norouziasas et al. [74]	✓	–	–	✓	–	✓	Block arrays	–
Alwi et al. [75]	–	✓	–	✓	–	–	Block arrays	–
Son et al. [76]	✓	–	–	✓	–	–	Block arrays	–
Hirose et al. [45]	–	–	✓	✓	–	–	Block arrays	–
Hirose et al. [77]	–	✓	–	✓	–	–	Block arrays	–
Ibrahim et al. [78]	–	✓	–	✓	–	–	Block arrays	–

*CFD* computational fluid dynamics, *WTE* wind tunnel experiment.

The investigation of wind flows in an urban environment is essential as the wind plays an important role in urban heat island formation, pollutant dispersion, and the embedded risk of wind. Additionally, studies on the urban wind environment aim to pinpoint areas where adversely high wind speeds might develop. While many studies have attempted this, most were based on steady-state velocity fields that were either derived using the CFD turbulence modeling approach [31–34] or detected with thermosensor sensors in WTEs [35–38]. The irregular layout of the urban morphology with the existence of high-rise buildings may influence the canopy layer wind and contribute to the strong wind occurrence around the high-rise building. As proven, unfavourable wind conditions might cause the operation of the new buildings to fail [16]. More strong wind events are expected to happen during the peak season of the monsoon season, in addition to the unpredictable effect of climate change. Thus, it is worthwhile to investigate the relationship between the canopy layer wind and building morphological parameters to understand the airflow characteristics. Wind profile has been used to describe the distribution of a (horizontal) mean wind speed at a vertical height. Lu et al. [39] suggested that looking into the vertical wind distributions would be beneficial for safety assessment or future improvement plans. Therefore, this study investigates the influence of wind at two different angles on high-rise buildings using a realistic geometry model within a tropical climate canopy height, utilising wind tunnel experiments (WTE). The implementation of the realistic geometry model using WTE distinguishes the present study from other related that are based either on a simplified model [12, 17, 40–45] or in computational fluid dynamics (CFD) application mode [46–50]. In addition, this study identified how several factors (downdraft effect, venturi effect, wake formation on the building’s leeward side, corner edge design, and canopy height) contribute to high wind speeds.

## Experimental methodology

This section describes how this study was implemented in detail, including the selected target site, wind-tunnel setup, measurement of vertical wind profiles, Reynolds number and the calculation of statistical parameters.

### Target site

An urban campus, Universiti Teknologi Malaysia Kuala Lumpur (UTMKL), has been selected as the target site of this study due to its location in a densely populated area near the centre of Kuala Lumpur, the capital city of Malaysia, making it a representative sample. Malaysia is a tropical country with a transition period between the northeast and southwest monsoons, which typically happens twice a year with frequent windstorms and a wind speed that can exceed 15.4 m/s [51], which may damage the buildings [52–55]. In fact, certain areas of the campus were damaged by strong winds during a storm [35].

In [35], the satellite image of the urban campus and the visual representation of the campus layout with respective building heights are depicted. The northern section of the campus mainly consists of low to medium-height buildings, and there are low-rise buildings in the vicinity of Residensi Tower (RT) of UTMKL. As mentioned, the central area of interest is how the high-rise buildings influence the wind distribution patterns within the urban canopy layer. In this context, only high-rise buildings are considered the target buildings. Two high-rise buildings were identified as having strong wind in their vicinity, RT (94 m) and MR (Menara Razak) (84 m).

### Wind-tunnel setup

Different wind tunnel laboratories may lead to mean wind speed and turbulence intensity variations [56]. Therefore, analysing an incoming flow that accurately resembles actual conditions is crucial before conducting any WTE. The closed-circuit wind tunnel at the laboratory of the Interdisciplinary Graduate School of Engineering Sciences (IGSES), Kyushu University, Japan, with dimensions of 8.0 m (length) × 1.5 m (width) × 1.0 m (height) and a turn table with a diameter of 1.28 m, which employed in previous studies [19, 35, 57] was used in this study. The scaled testing models (1:750) ensure a capped blockage ratio of less than 3% to prevent wind tunnel wall effects [58] were prepared according to the exact building shapes and layout. However, larger model will increase the blockage and speed up the flow. Hence, imitating an accurate depiction of the actual urban environment to avoid overlooking the presence of noteworthy buildings [59]. However, this study does not consider intricate geometry and specific characteristics of structures, such as roof designs, windows, balconies, or openings. Adopting the standard practice [60], landscape or vegetations are omitted.

**Fig. 1:**
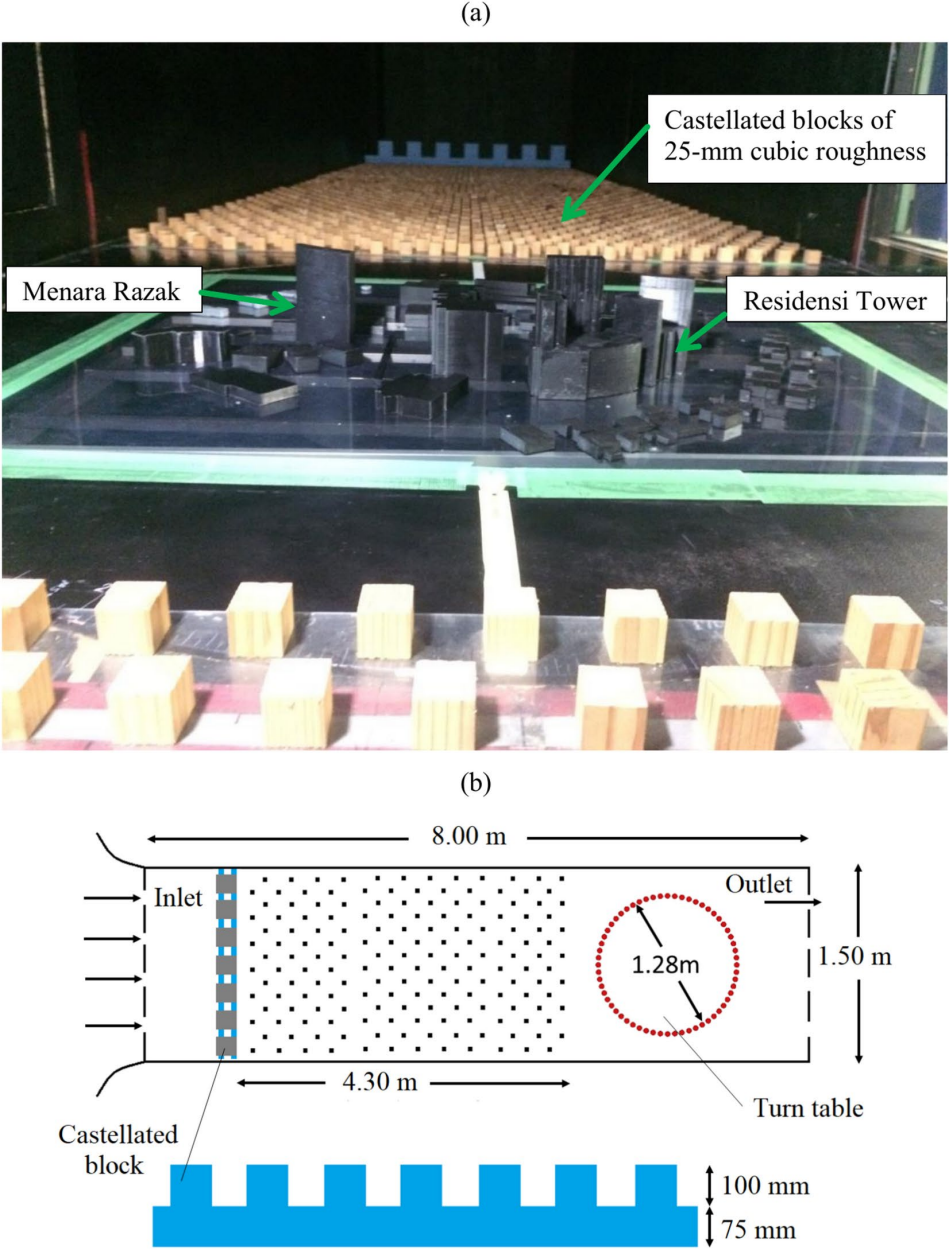
Wind tunnel experiment layout plan (**a**). The entire wind tunnel consists of castellated blocks and testing building models (**b**). The castellated blocks are built from two rows of 2D arrays (75 mm in height) and added on top with 100 mm cubic.

A castellated block and staggered layout flooring (4.30 m) consisting of 25-mm cubic roughness elements with a packing density of 17% reproduced the approaching wind profile. Figure 1 shows the layout plan of the WTE, where roughness setup resulted in the vertical profiles of approaching wind that satisfies a power law index of 0.23 and falls within the suburban terrain category. The simulation conditions closely resemble the target site, which is located 5 km away from the Kuala Lumpur city centre.

### Measurement of vertical wind profiles within the canopy layer

**Fig. 2:**
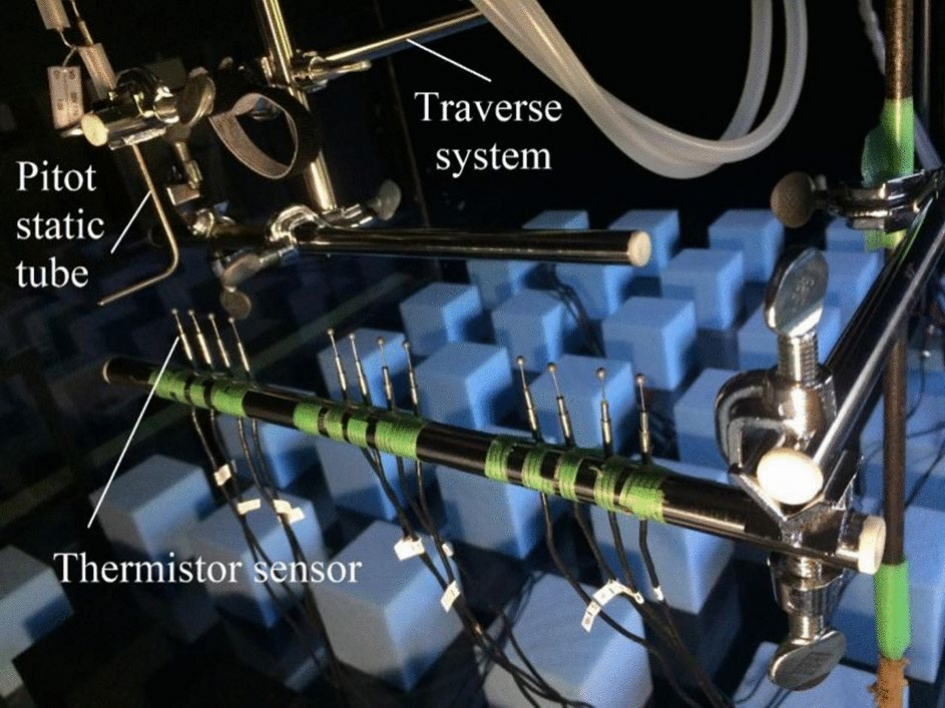
Calibration setup for thermistor sensors and pitot static tube.

Each target building (MR and RT) in the urban campus was examined under the two selected wind directions, 22.5° (west–south) and 202.5° (north–east) since higher wind speeds were observed near these buildings during the previous test done on a pool of 16 different wind conditions. An I-type hotwire anemometer (HWA) (KANOMAX, Model 0251R-T5) was used to measure the turbulent wind speeds around the buildings, positioned at a height of 5 mm and connected to a support probe (KANOMAX, Model 0102). The bias errors of the HWA were 0.2% and 2% for mean and turbulent velocities, respectively [61]. Moreover, the turbulence intensity is considered to be about 0.2 to 0.1%, as reported in H’ng et al. [35]. A limitation of utilising this I-type HWA is that it only measures the wind speed determined by the two velocity components perpendicular to the HWA, as the probe is installed with the wire aligned in the y-direction (spanwise direction) as shown in Fig. 2. Hence, the influence of the spanwise velocity could not be accurately determined. Consequently, the impact of the wind directions was not fully considered. Due to the possibility of a different flow response around high-rise buildings in realistic conditions compared to isolated blocks studied previously, this measurement focuses on the strong wind effects caused by the target high-rise buildings within the canopy layer. The spanwise uniformity within the wind tunnel test section is confirmed by comparing the wind speed at different heights. The uniformity of the flow is then confirmed with an acceptable standard deviation of below 0.1.

**Fig. 3:**
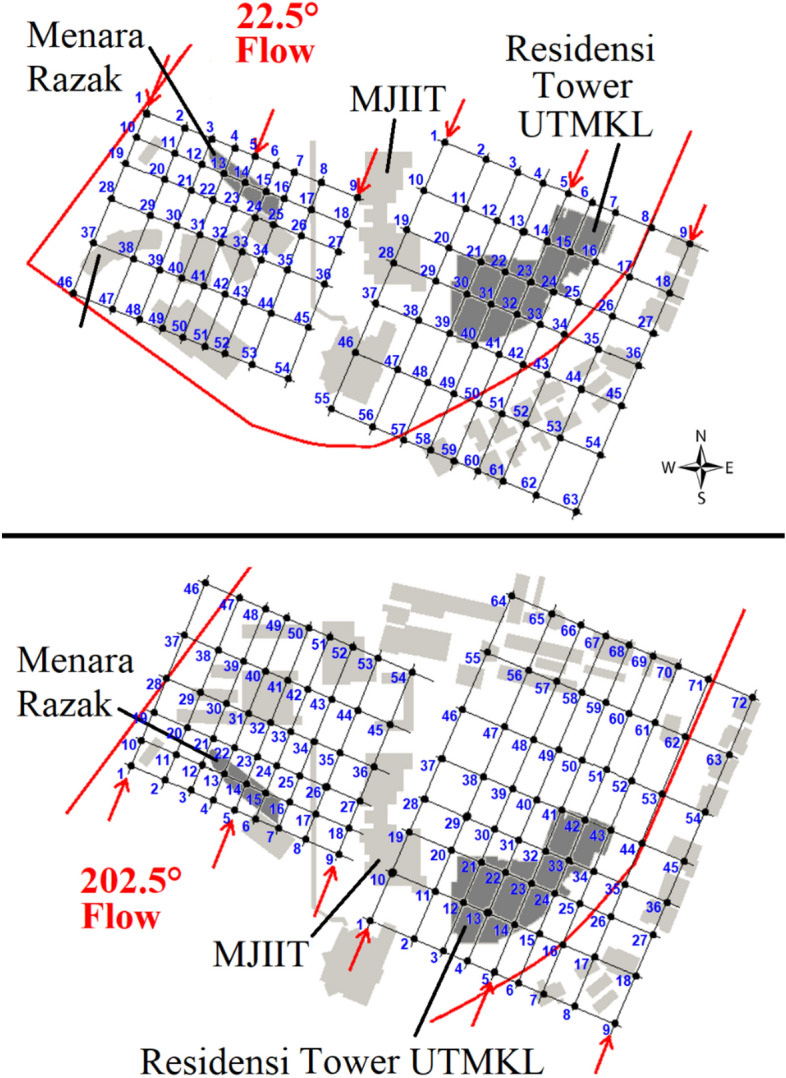
The numbers show the measurement points for the respective incident wind directions of 22.5° (west–south) and 202.5° (north–east), measuring from 5 mm and extended vertically to 230 mm (for MR building) and 250 mm (for RT building). The two target buildings (Menara Razak and Residensi Tower) are shaded with grey colour.

The measuring height of 5 mm corresponded to a scaled pedestrian level of 3.75 m, the lowest height possible, due to the mounting limitation of the traverse system with the hotwire probe. Despite being at a height greater than the typical pedestrian level (1.5 m and 2 m), the analysis focused on the wind speed characteristics that contribute to the formation of turbulent airflow within the canopy layer and at pedestrian level. To confirm whether the measurement points are sufficient to cover the regions that might be affected by the high-rise buildings, the measurement plan included the area with two times and 1.5 times the height of the targeted building at vertical and horizontal planes, respectively. Table 2 shows the total number of measurement points at the horizontal plane for MR and RT buildings under the two wind directions. The black dots in Fig. 3 represent the measurement points. The streamwise wind speed under the two selected wind directions was measured vertically at each measuring point with respective elevations. The built-in traverse system controlled the position of the HWA, enabling a precise movement throughout the experiment. The measurement starts at 5 mm from the wind tunnel ground and continues to the highest measuring point at 230 mm and 250 mm for MR and RT, respectively.

**Table 2:** The total number of measurement points at the horizontal plane for both targeted buildings, namely Menara Razak (MR) and Residensi Tower of UTMKL (RT), under wind directions of 22.5° and 202.5°. All measurement points are represented by black dots shown in Fig. 3.

Target building	MR		RT	
Wind direction	22.5°	202.5°	22.5°	202.5°
Building height in the wind tunnel (mm)	112		125	
Distance covered vertically (mm)	224		250	
Distance covered horizontally (mm)	168		188	
No. of measurement points	54	54	63	72

Each measurement point (black dots) is numbered accordingly, as shown in Fig. 3, to better observe the effects of target buildings towards strong winds on the X-plane (streamwise direction) and the Y-plane (spanwise direction) at two incident wind directions, 22.5° and 202.5°. For instance, the sequence numbers for the X-plane (streamwise direction) at 22.5° for the MR building are 1, 10, 19, 28, 37, and 46. Meanwhile, the sequence numbers for the Y-plane (spanwise direction) at 22.5° for the RT building are 1, 2, 3, 4, 5, 6, 7, 8, and 9.

### Reynolds number and statistical parameters

The statistical characteristics of turbulent flows were observed using wind speed and the frequency of 1000 Hz for 30 s. At the reference height *z*_*ref*_ of 500 mm where the inlet flow is undisturbed by the underlying roughness in the test section, the reference wind speed was measured by the Pitot tube. This height is fixed to ensure that the reference velocity is fixed or within a small range; the measured reference velocity is 6.3 < *u*_*ref*_ < 6.5 m/s. The details of the wind tunnel experiment can be referred in the previous study of H’ng et al. [35]. The resulting Reynolds number is the range of 21.0 × 10^4^ < *Re* (= *z*_*ref*_*u*_*ref*_/*ν*) < 21.7 × 10^4^ where *ν* ~ 1.5 × 10^−5^ m^2^/s is the kinematic viscosity of air. In terms of the average building height (*h*_*av*_ = 12.6 mm), the corresponding Reynolds number is in the range of 5.3 × 10^3^ < *Re* (= *h*_*av*_*u*_*ref*_/*ν*) < 5.5 × 10^3^. In addition, the building height Reynolds number is *Re*_*hav*_ = *h*_*av*_*u*_*hav*_/*ν *~ 2.3 × 10^3^ [35]. Each measurement was repeated three times, and the ensembled average was taken. Results indicate that the coefficient variation among the three trials at the same position for the mean values averages between 1.24 and 1.74%.

The vertical profiles of each measurement point were used to generate a distinctive overview of the effects of targeted buildings towards wind speeds within the canopy layer in both horizontal and lateral directions using colour contour maps. Some parameters were identified for further investigation into the influence of those targeted buildings. Specifically, the distributions for vertical profiles of mean wind speed (*u*), root mean square (*u*_*rms*_), and skewness (*SK*) within the canopy layers were more representative for depicting the wind distribution of the interest study area, especially under strong wind conditions. The respective calculations are expressed in Eqs. (1) and (2).

1 $${u}_{rms}=\sqrt{\frac{1}{N}\sum_{i=1}^{N}{\left({u}_{i}-u\right)}^{2}}$$

2 $$SK=\frac{\frac{1}{N}\sum_{i=1}^{N}{\left({u}_{i}-u\right)}^{3}}{{u}_{rms}}$$

where *u*_*i*_ is the instantaneous wind speed and *N* is the number of measured data. Both *u* and *u*_*rms*_ are involved in eddy transport; the wind speed fluctuation within the flow can be observed through *u*_*rms*_, and SK may help to grasp how often the strong wind occurs. Each target building contains presentations of the distributions for both wind directions. Nonetheless, only observations that visualise the wind effects of the target buildings will be discussed in this context.

## Results and discussion

This section provides a detailed analysis of measured wind speeds and statistical parameters obtained from the wind tunnel experiments. MATLAB software generated the contours of the wind at the wake of buildings. The effects of wind incidences on two high-rise buildings, Menara Razak (MR) and Residensi Tower (RT), are thoroughly discussed.

### Effect of substantial wind incidence at 22.5° on Menara Razak

**Fig. 4:**
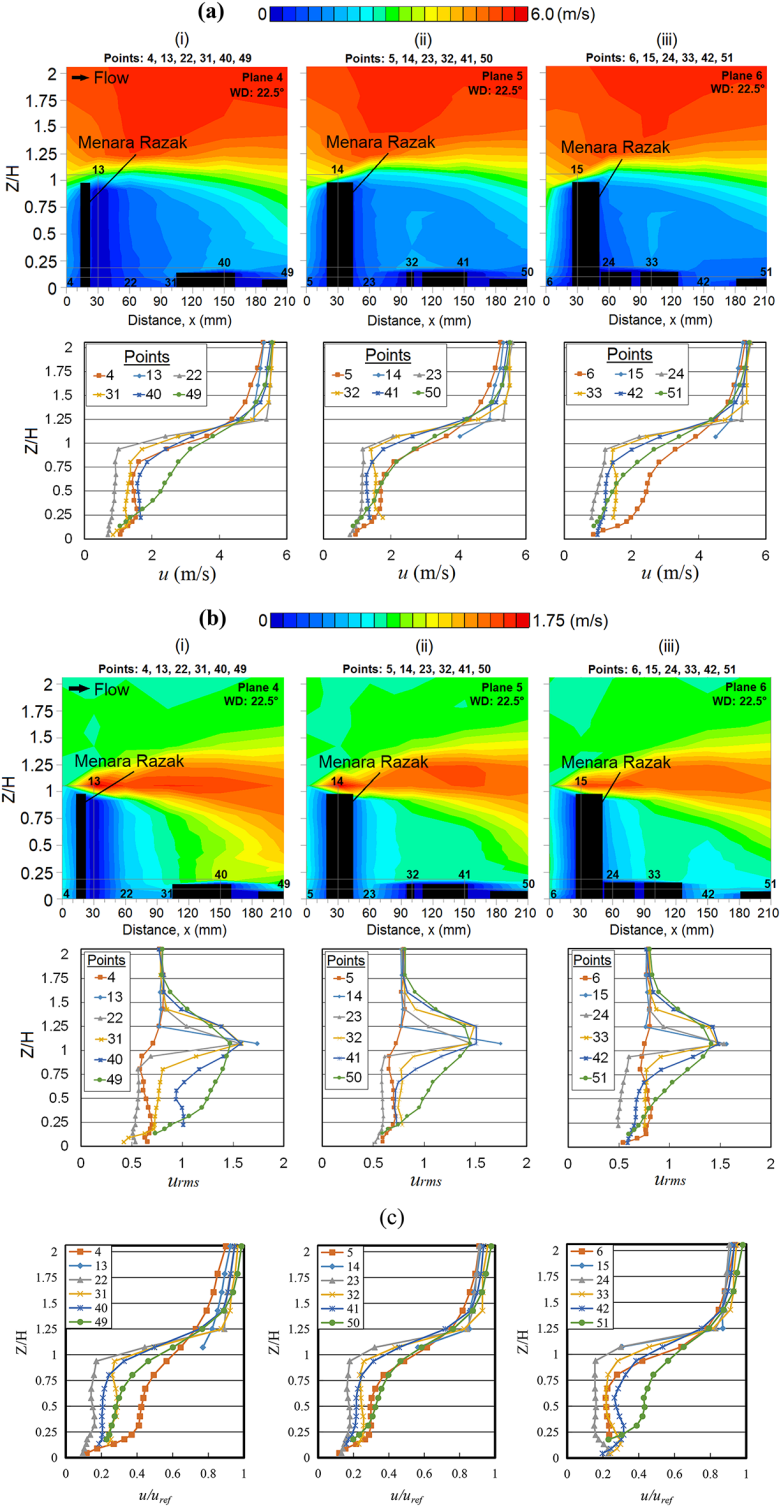
(**a**) Vertical profiles of the mean wind speed ‘*u*’ (**b**) Vertical profiles of the root mean square velocity ‘*u*_*rms*_’ and (**c**) Speed-up factor of wind at respective X-plane(s) (streamwise directions) within the canopy layer with Menara Razak (MR) as the target building under the wind direction (WD) of 22.5°: (i) Plane 4; (ii) Plane 5 and (iii) Plane 6. The ordinate *Z*/*H*: *Z* is the canopy layer in the wind tunnel, normalized by *H*, the height of the target building model (112 mm).

Turbulent winds are expected at the leeward of the building as flow recirculation occurs and vortices are formed under a lower pressure zone [62]. Although the variation of *u* at the leeward of the MR building is not significant, the increment of *u* is still noticeable. Corresponding to this, the wind fluctuations of vertical profiles of *u*_*rms*_ can be clearly shown in Fig. 4b, where vigorous strong winds may occur at the lower ground behind the MR building. In addition, a highly turbulent flow recirculation was observed near the building’s roof edge [63], travelling downstream, and dissipating. A plume-liked shape is clearly shown in Fig. 4a, b. Moreover, Fig. 4c shows the vertical profiles of the speed-up factor at several positions concerning the high-rise building MR. The profiles are represented by the normalized mean wind speed with the reference velocity to quantify the amplification of wind speed at different positions. The results indicate the same pattern; the wind speed gradual increases as the position increases from the high-rise building due to the wake flow in its downstream region.

**Fig. 5:**
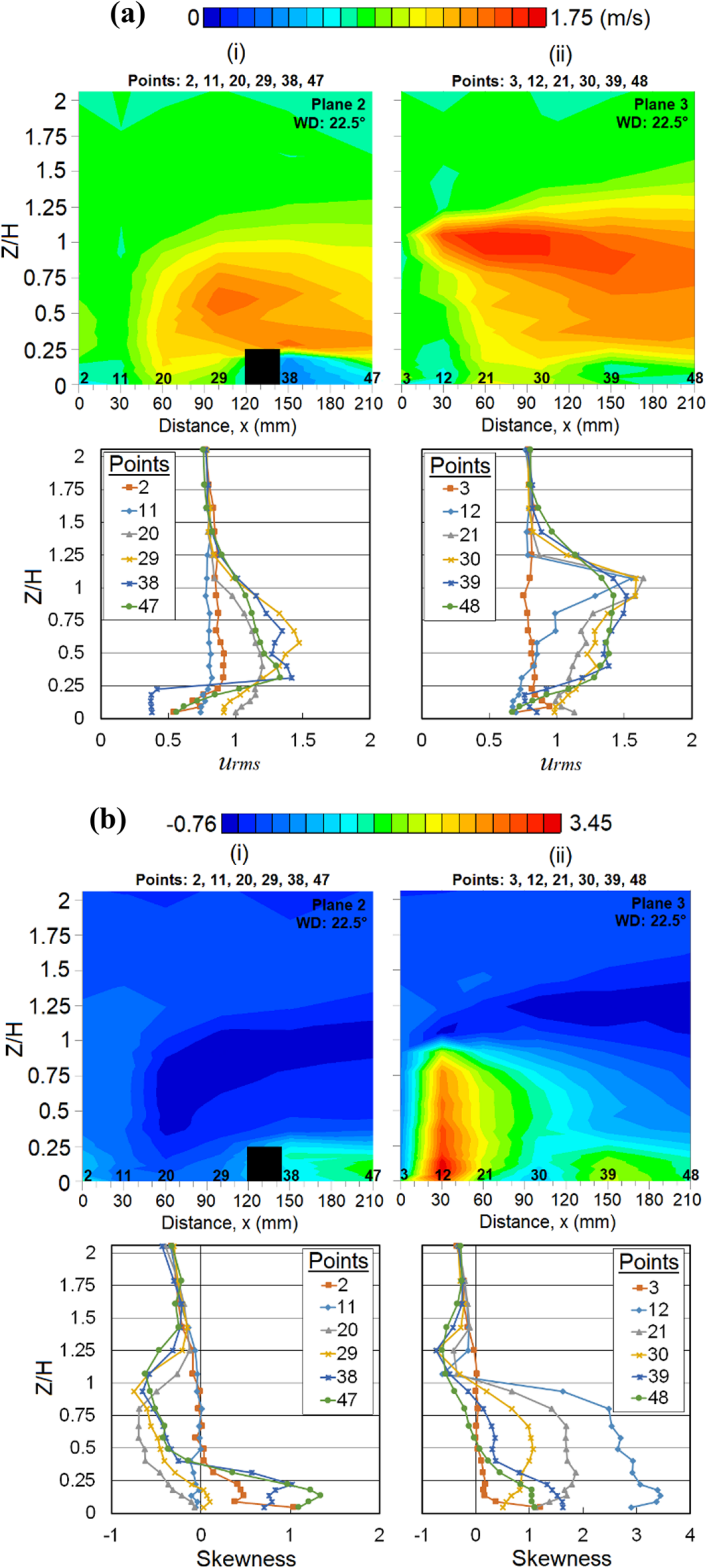
Vertical profiles of (**a**) the root mean square, *u*_*rms*_ and (**b**) skewness (SK) at respective X-plane(s) (streamwise directions) within the canopy layer with Menara Razak (MR) as a target building under the wind direction (WD) of 22.5°: (i) Plane 2 and (ii) Plane 3. The ordinate *Z*/*H*: *Z* is the canopy layer in the wind tunnel, normalized by *H*, the height of the target building model (112 mm).

Speed-up winds were spotted near the top corner of the MR building (at points 20, 21 and 30). These winds are supported by comparing the vertical profile distribution of *u*_*rms*_ Fig. 5a where the fluctuation and dispersion of wind occurs, while the low SK distribution at those regions is shown in Fig. 5b indicating where the strong winds occur. Low *u*_*rms*_ values were found at point 12 Fig. 5a(ii) where the flow separation occurs at the building corner. On the other hand, the slightly round-shaped corner of the MR building imposes less drag on the flow (as opposed to sharp corners), thus amplifying the winds [62]. Considering that the HWA may underpredict the exact mean wind speed in the recirculation region, the strong wind observed in these regions should have been given some attention as a precaution [30].

**Fig. 6:**
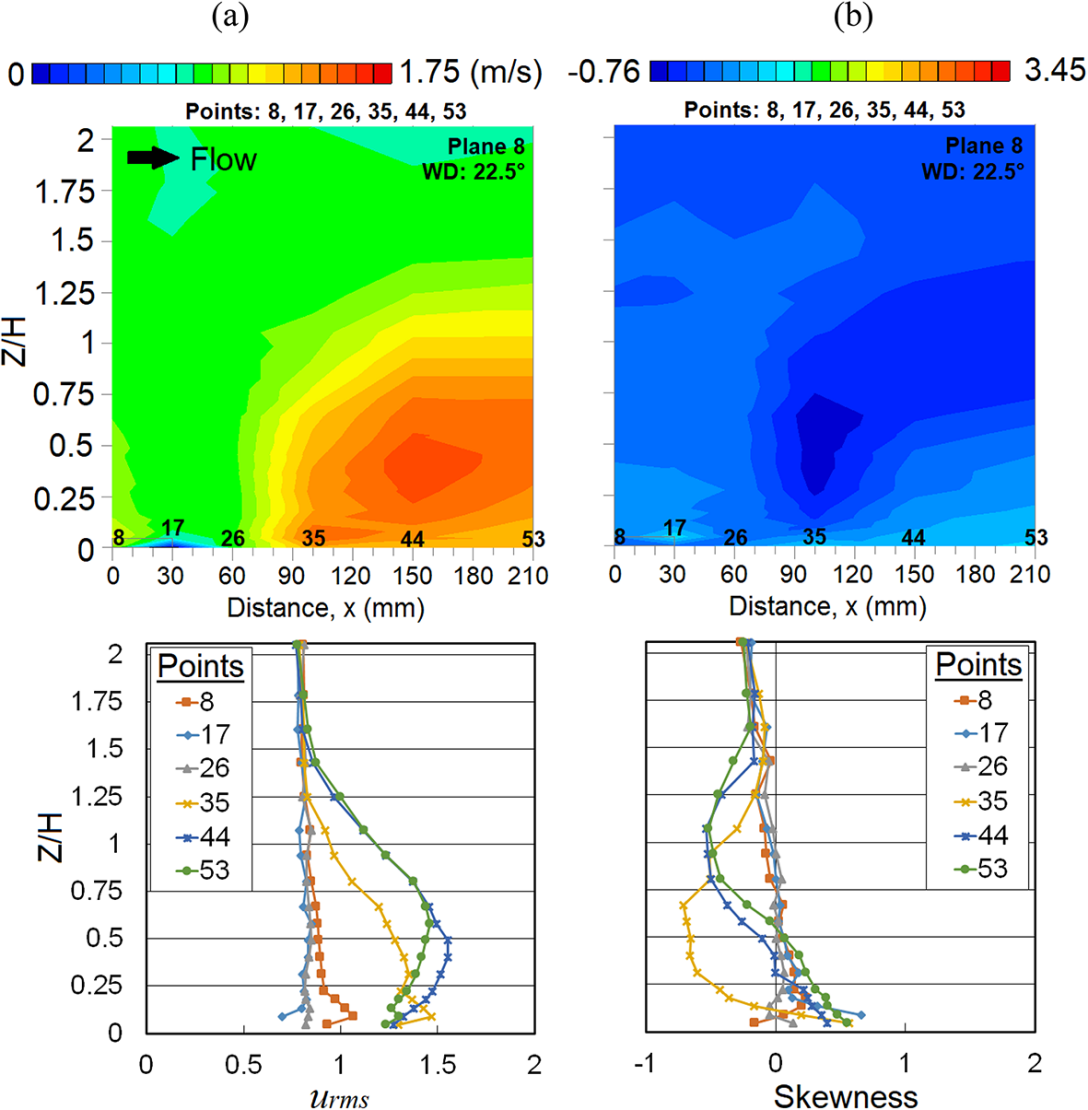
Vertical profiles of (**a**) the root mean square, *u*_*rms*_ and (**b**) skewness (SK) at the X-plane: Plane 8 (streamwise direction) within the canopy layer with Menara Razak (MR) as the target building under the wind direction (WD) of 22.5°. The ordinate *Z*/*H*: *Z* is the canopy layer in the wind tunnel, normalized by *H*, the height of the target building model (112 mm).

In addition, the occurrence of strong winds at a further downstream distance is due to the influence of the MR building at the leeward corner region (at points 26, 35 and 44). The vertical profile distribution of *u*_*rms*_ was found higher at a lower canopy layer, indicating that there are more wind fluctuations taking place (Fig. 6a), while the decrease in the distribution of SK indicates how often the strong wind occurs within the regions due to the influence of the MR building; the occurrences of strong winds can be interpreted from Fig. 6b. The large adverse pressure on the leeward side of the building may have contributed to the existence of the recirculation region [30]. The vortices formed might have travelled and led to the strong winds towards a further distance up to *H*, where *H* is the height of the target MR building.

**Fig. 7:**
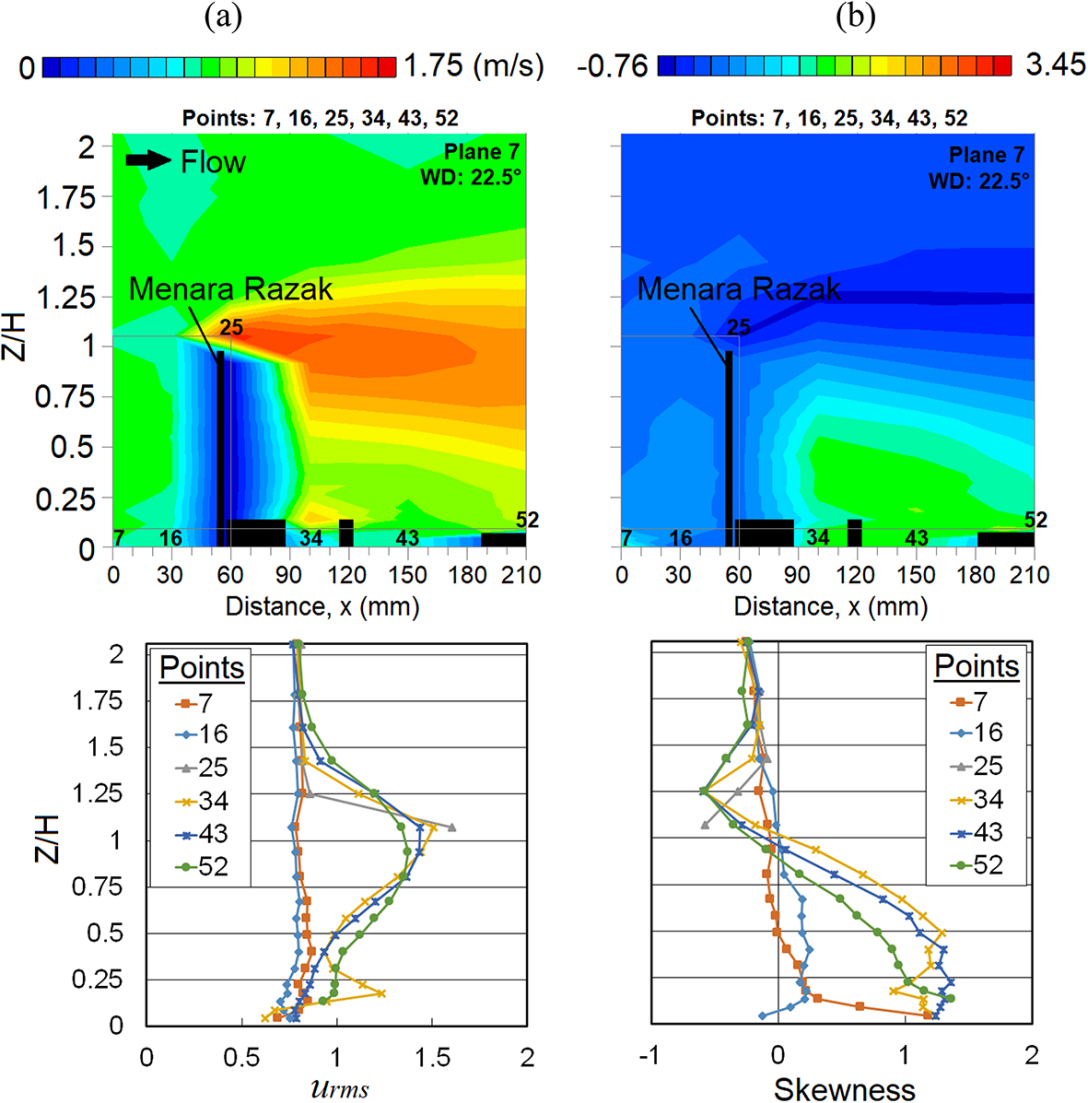
Vertical profiles of (**a**) the root mean square, *u*_*rms*_ and (**b**) skewness (SK) at the X-plane: Plane 7 (streamwise direction) within the canopy layer with Menara Razak (MR) as the target building under the wind direction (WD) of 22.5°. The ordinate *Z*/*H*: *Z* is the canopy layer in the wind tunnel, normalized by *H*, the height of the target building model (112 mm).

Due to the existence of the assembled buildings next to the MR building, the leeward flow at points 34 and 43 is deflected as per the distribution of *u*_*rms*_ shown in Fig. 7a. However, there is a higher fluctuation of wind near the rooftop of the building located above point 34. With the combination of the downdraught effect and the flow separation near the corner building (at point 16), the negatively skewness distribution (see Fig. 7b) at a lower ground level indicates that the flow is leaning towards a higher wind speed.

### Effect of strong wind incidence at 202.5° on Menara Razak

**Fig. 8:**
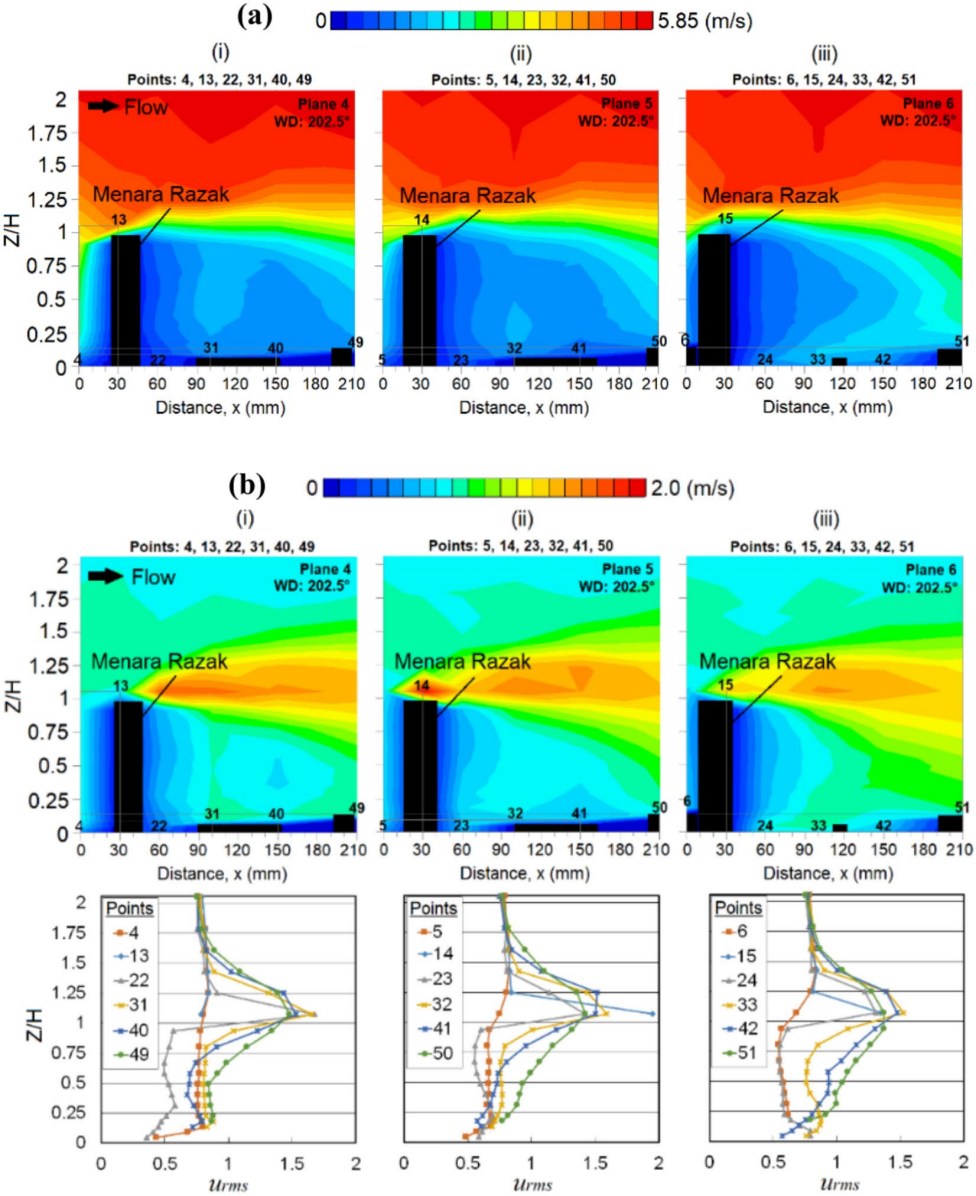
Vertical profiles of (**a**) the mean wind speed, ‘*u*’ and (**b**) the root mean square speed ‘*u*_*rms*_’ at the respective X-plane(s) (streamwise directions) within the canopy layer with Menara Razak (MR) as the target building under the wind direction (WD) of 202.5°: (i) Plane 4; (ii) Plane 5 and (iii) Plane 6. The ordinate *Z*/*H*: *Z* is the canopy layer in the wind tunnel, normalized by *H*, the height of the target building model (112 mm).

At the wind direction of 202.5°, the vertical profiles of *u* near the leeward regions of the MR building in the streamwise direction are shown in Fig. 8a. Corresponding to this, the *u*_*rms*_ distributions depicted in Fig. 8b show the fluctuation of velocities, indicating that the regions at the distance within the minimum coverage of *H* (where *H* is the height of the target MR building) may experience strong winds.

Although a higher *u* is expected near the windward of the MR building, this phenomenon is hardly spotted in Fig. 8a. This can be explained by the interaction of combined downdraught and approaching wind which creates a weak wind zone at the lower ground of the windward building [62].

**Fig. 9:**
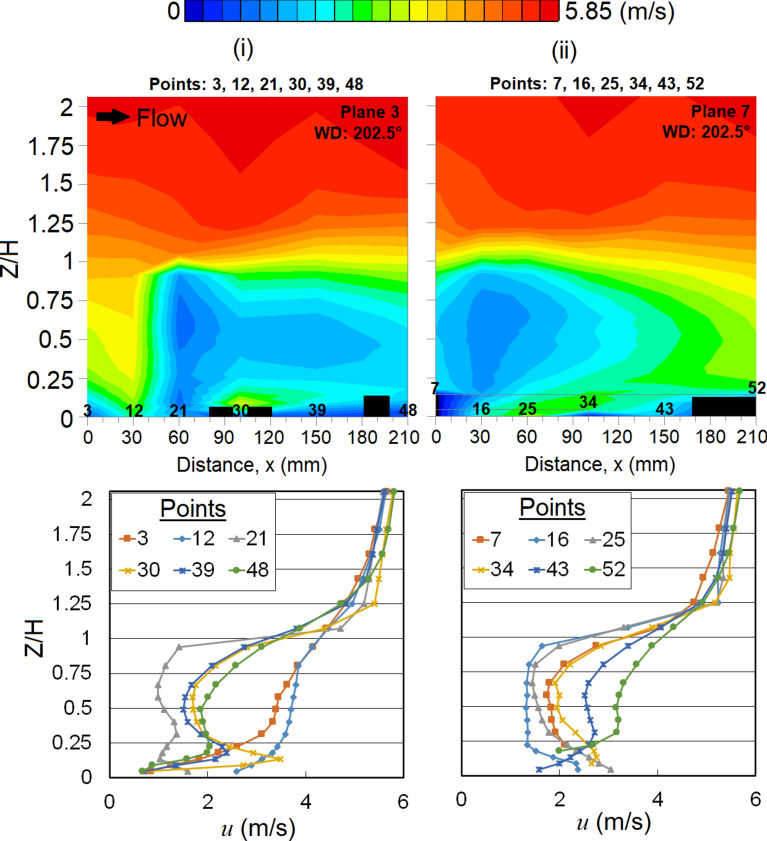
Vertical profiles of the mean wind speed, *u* at respective X-plane(s) (streamwise directions) within the canopy layer with Menara Razak (MR) as the target building under the wind direction (WD) of 202.5°: (i) Plane 3 and (ii) Plane 7. The ordinate *Z*/*H*: *Z* is the canopy layer in the wind tunnel, normalized by *H*, the height of the target building model (112 mm).

**Fig. 10:**
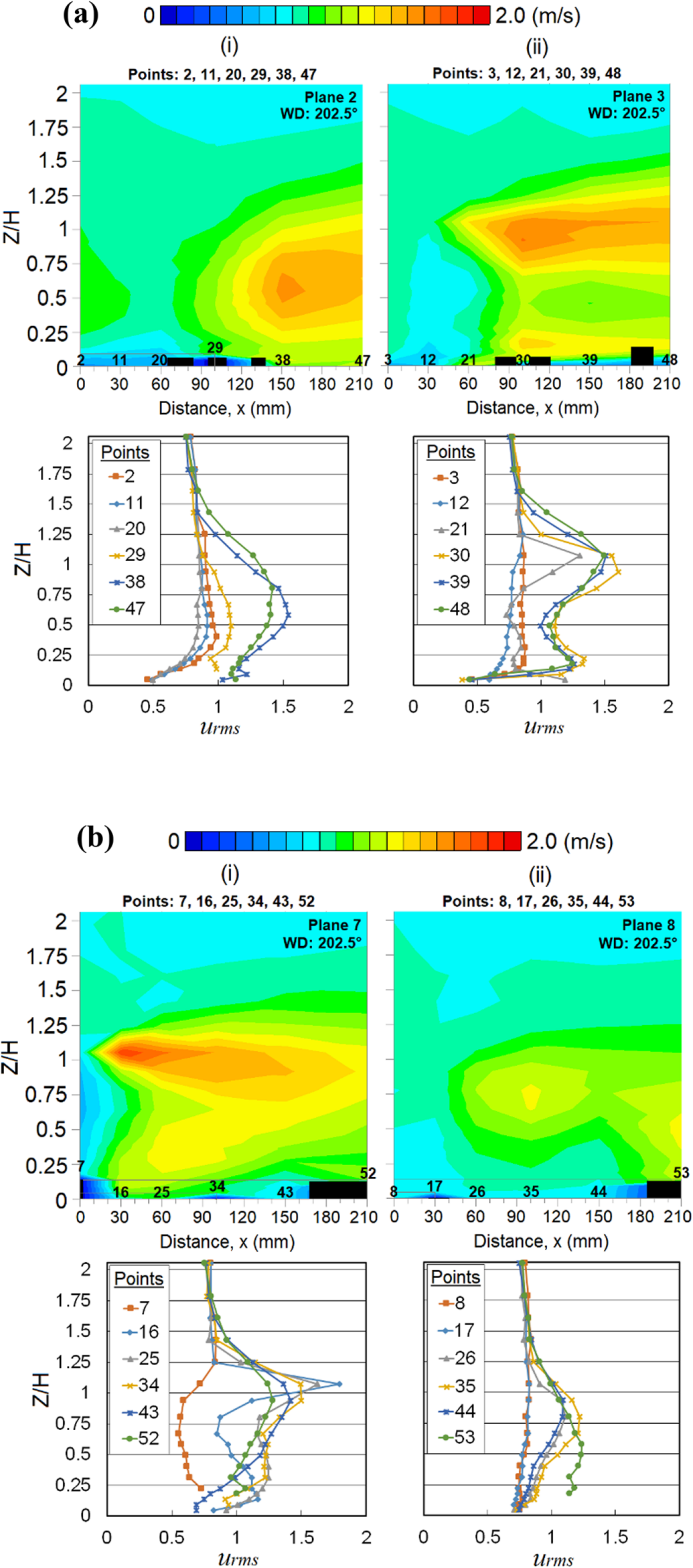
Vertical profiles of the root mean square, *u*_*rms*_ near (**a**) left corner and (**b**) right corner of Menara Razak (MR) at respective X-plane(s) (streamwise directions) within the canopy layer under the wind direction (WD) of 202.5°: (**a**, i) Plane 2; (**a**, ii) Plane 3 (**b**, i) Plane 7 and (**b**, ii) Plane 8. The ordinate *Z*/*H*: *Z* is the canopy layer in the wind tunnel, normalized by *H*, the height of the target building model (112 mm).

Meanwhile, variations of *u* are found at both leeward corners of the MR building (Planes 3 and 7) which can be seen from the vertical profiles of *u* shown in Fig. 9. The flows at these regions are highly turbulent and the vortices are spreading further in the spanwise direction, as shown in the vertical profiles of *u*_*rms*_ in Fig. 10a, b.

### Effect of strong wind incidence at 22.5° on Residensi Tower

**Fig. 11:**
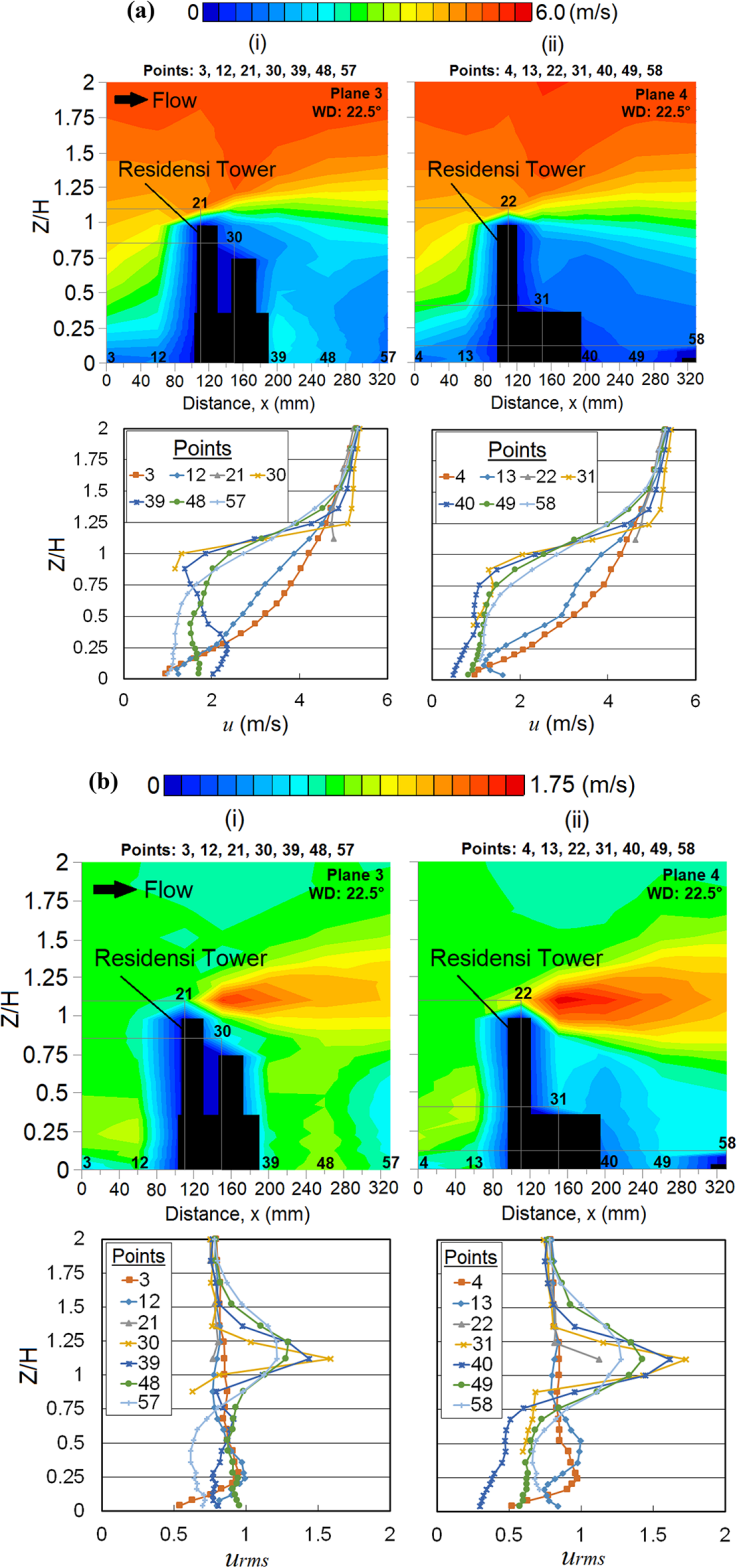
(**a**) Vertical profiles of the mean wind speed *u* and (**b**) vertical profiles of the root mean square *u*_*rms*_ at respective X-plane(s) (streamwise directions) within the canopy layer with Residensi Tower (RT) as the target building under the wind direction (WD) of 22.5°: (i) Plane 3 and (ii) Plane 4. The ordinate *Z*/*H*: *Z* is the canopy layer in the wind tunnel, normalized by *H*, the height of the target building model (125 mm).

At the wind direction of 22.5°, a higher mean wind speed *u* can be observed near the windward of the RT building at the lower ground level, especially at points 12 and 13. Vertical profiles of *u* are shown in Fig. 11a. The elevation of wind speeds is mostly influenced by the L-shape of the RT building, where the induced wind from the edge corner at point 5 combined with the approaching flow may have further increased the downdraught effect. In addition, the *u*_*rms*_ distribution in Fig. 11b shows that the flows at points 12 and 13 are more turbulent.

**Fig. 12:**
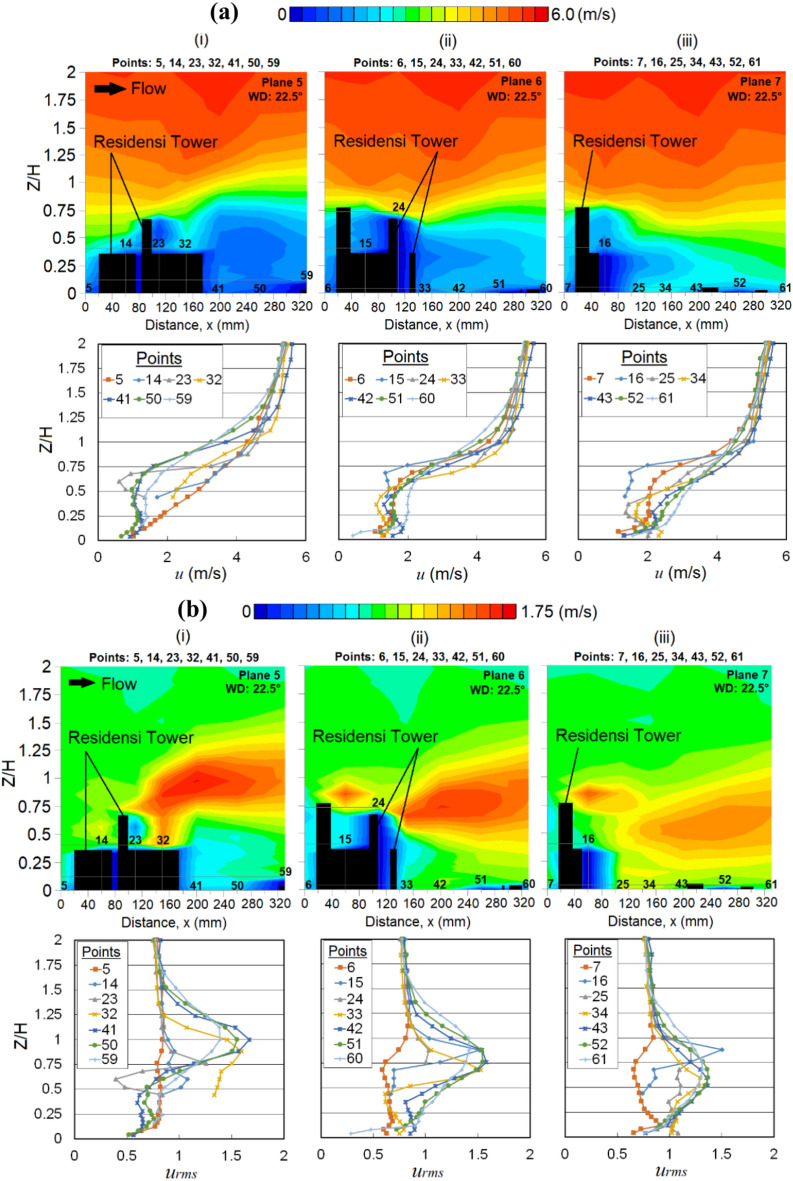
Vertical profiles of (**a**) the mean wind speed, *u* (**b**) the root mean square, *u*_*rms*_ at respective X-plane(s) (streamwise directions) within the canopy layer with Residensi Tower (RT) as the target building under the wind direction (WD) of 22.5°: (i) Plane 5; (ii) Plane 6 and (iii) Plane 7. The ordinate *Z*/*H*: *Z* is the canopy layer in the wind tunnel, normalized by *H*, the height of the target building model (125 mm).

The curved design of the RT building corner somehow contributes to most of the wind amplifications near the leeward and building corner regions. Although sheltering effect occurs behind the RT building at points 40 (Fig. 11a (ii)) and 41 (Fig. 12a(i)), where high fluctuation of wakes might not be found, the fluctuation patterns are still noticeable (Fig. 12b shows the *u*_*rms*_ distributions). The wakes extending downstream at a minimum of 1.5*H*, where *H* is the height of the target RT building are shown in Fig. 12b (iii); this induced strong wind due to the podium structure potentially affects the nearby community over a larger region with low-rise buildings [64]. This somehow provides us with an early alarm for preparing the remediation and precautionary measures for potentially dangerous wind gusts in the future.

Suppose, the podium structure is believed to serve the purpose of shielding the pedestrians from strong winds at ground level [65], yet, the wind environment at the podium shall not be neglected, as some of the outdoor facilities are built for recreational purposes. In this case, strong winds are spotted at the podium of the RT building (at point 32; see Fig. 12a(i)). Figure 12b(i) shows that the turbulent flows exist near the gaps between the two high blocks (Block 1 and Block 3) at the podium of the RT building. At a higher canopy layer (podium level of 37 m), the wind speed of the approaching flow is higher. When the wind flow passes through the gap between the blocks, the speed-up wind tends to be vigorous. Thus, this induced strong wind at the podium level might pose some risks to the communities.

**Fig. 13:**
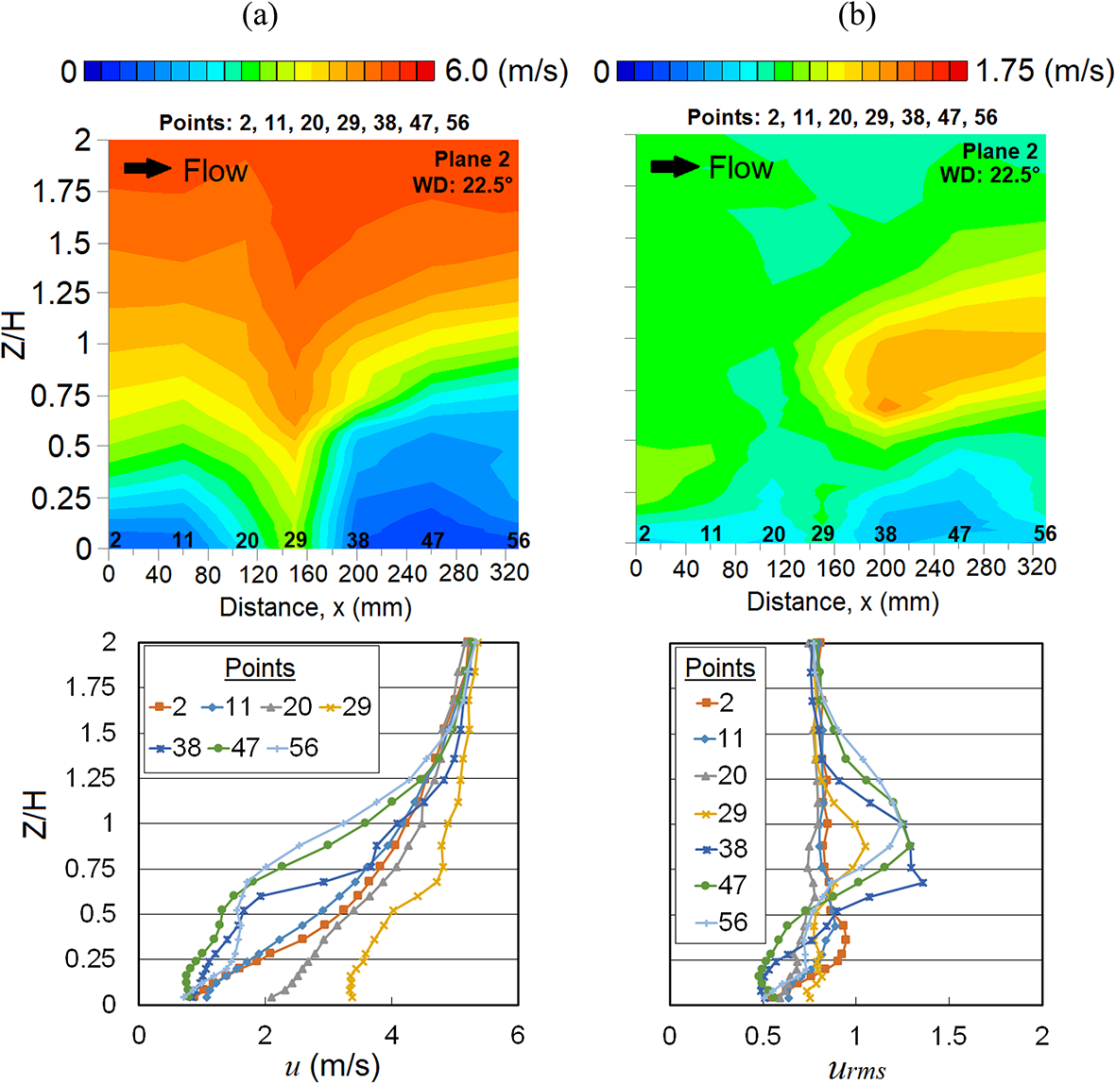
Vertical profiles of (**a**) the mean wind speed, *u* and (**b**) the root mean square, *u*_*rms*_ at X-plane: Plane 2 (streamwise directions) within the canopy layer with Residensi Tower (RT) as the target building under the wind direction (WD) of 22.5°. The ordinate *Z*/*H*: *Z* is the canopy layer in the wind tunnel, normalized by *H*, the height of the target building model (125 mm).

Meanwhile, the occurrence of Venturi effect is found at point 29 where the wind converges and flows through the two high-rise (MJIIT and RT) buildings. The speed-up winds are clearly shown from the vertical profile distribution of *u* in Fig. 13a and its *u*_*rms*_ distribution is shown in Fig. 13b.

**Fig. 14:**
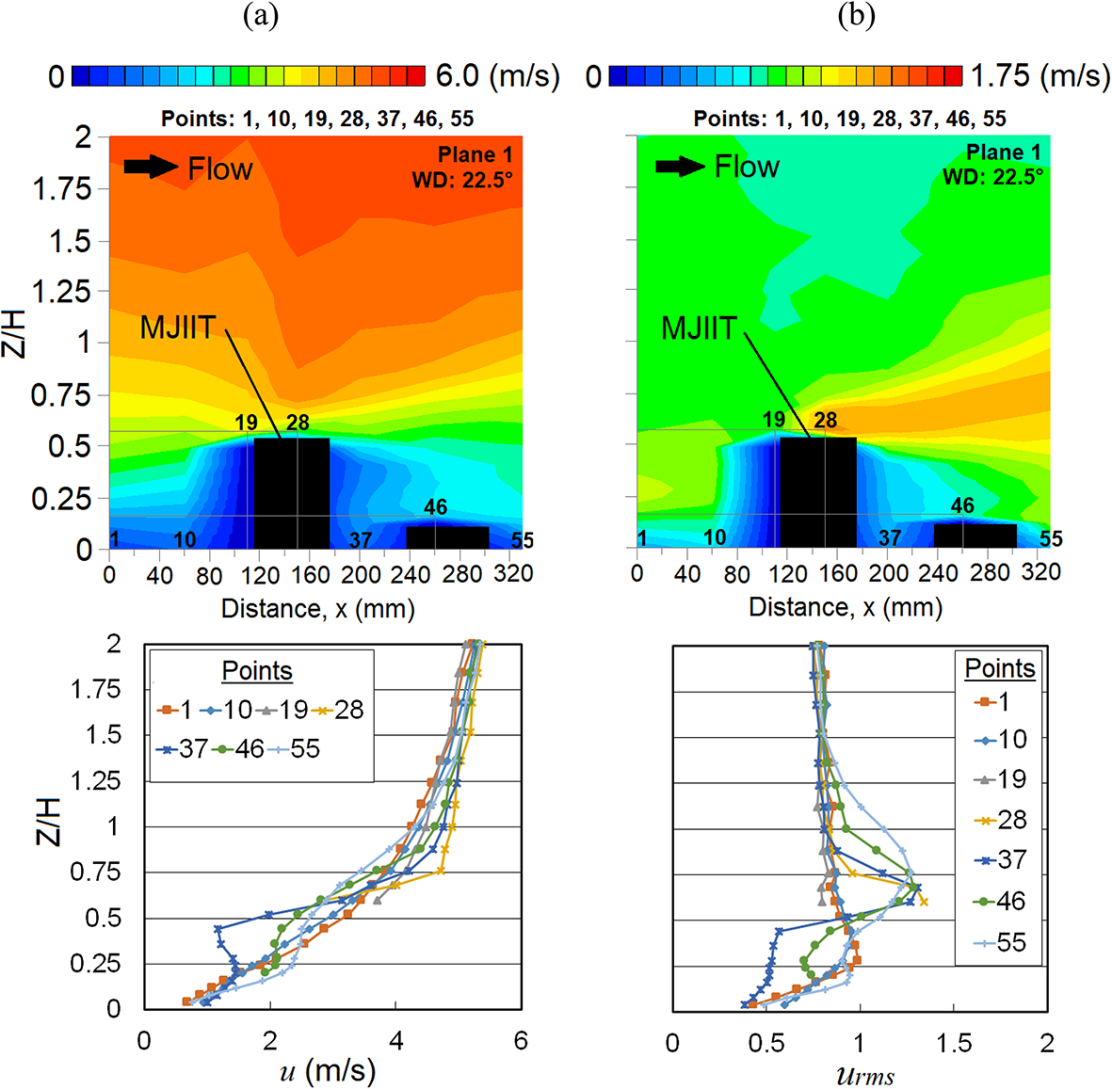
Vertical profiles of (**a**) the mean wind speed, *u* and (**b**) the root mean square, *u*_*rms*_ at X-plane: Plane 1 (streamwise directions) within the canopy layer with Residensi Tower (RT) as the target building under the wind direction (WD) of 22.5°. The ordinate *Z*/*H*: *Z* is the canopy layer in the wind tunnel, normalized by *H*, the height of the target building model (125 mm).

Referring to both vertical profiles and distributions of *u* and *u*_*rms*_ shown in Fig. 14, at point 37, the separation flow due to the MJIIT building combines with the speed-up approaching wind induced by the Venturi effect at point 29. These combined winds move towards the canyon and interact with the downstream building causing the upward wind flow [66].

### Effect of strong wind incidence at 202.5° on Residensi Tower

**Fig. 15:**
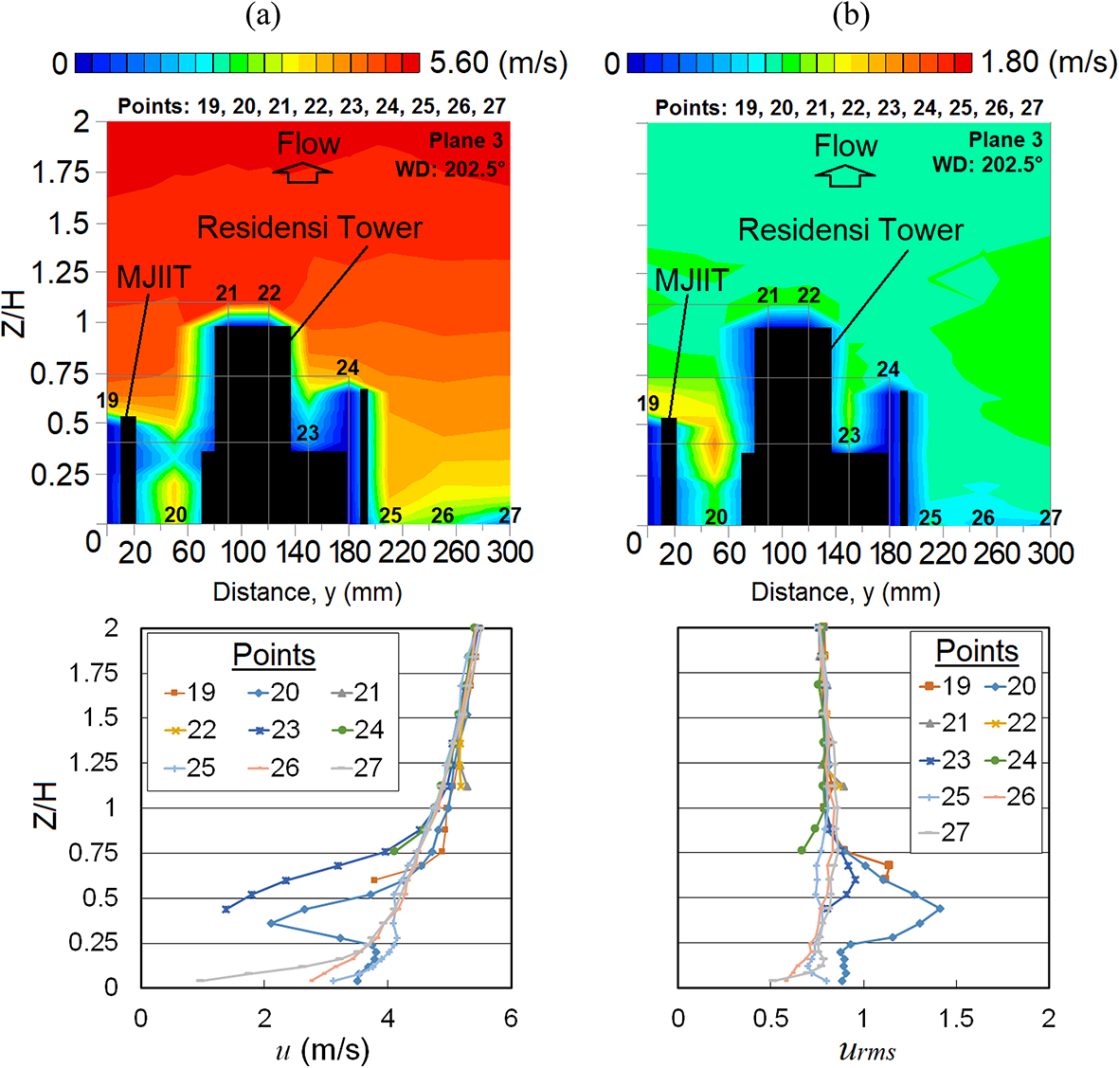
Vertical profiles of (**a**) the mean wind speed, *u* and (**b**) the root mean square, *u*_*rms*_ at Y-plane: Plane 3 (spanwise directions) within the canopy layer with Residensi Tower (RT) as the target building under the wind direction (WD) of 202.5°. The ordinate *Z*/*H*: *Z* is the canopy layer in the wind tunnel, normalized by *H*, the height of the target building model (125 mm).

A high *u* is spotted near the ground at point 20, located near the edge corner of the building right after the gap between the two high-rise buildings (MJIIT and RT). When the upstream wind passes through the passage, the wind flow converges and amplifies the wind speed, causing the Venturi effect. With the combination of the Venturi effect and the speed-up wind due to the edge corner of the building, the intensification of *u* at this point is reasonable and the flow is more turbulent as presented in both vertical profiles and distributions of *u* and *u*_*rms*_ shown in Fig. 15.

**Fig. 16:**
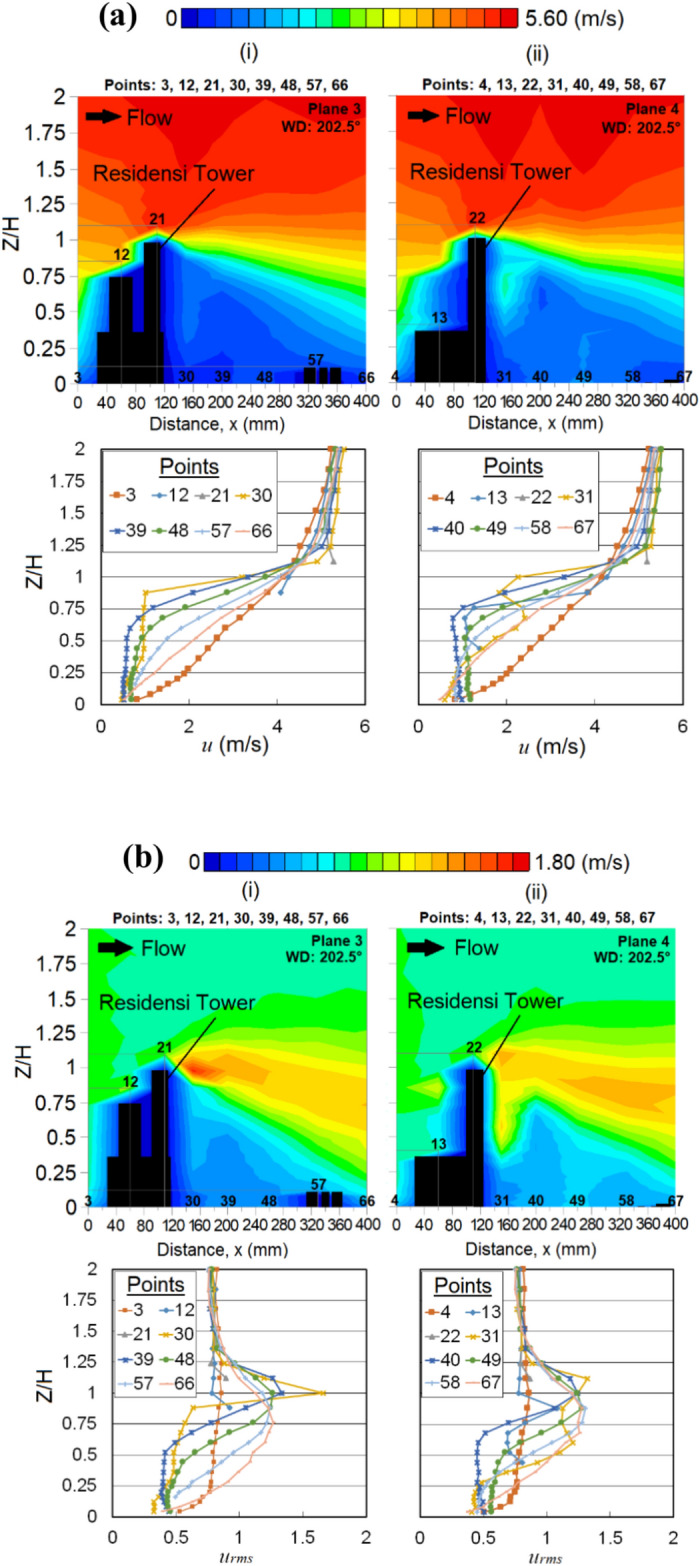
Vertical profiles of (**a**) the mean wind speed, *u* and (**b**) the root mean square, *u*_*rms*_ of Residensi Tower (RT) building at respective X-plane(s) (streamwise directions) within the canopy layer under the wind direction (WD) of 202.5°: (i) Plane 3 and (ii) Plane 4. The ordinate *Z*/*H*: *Z* is the canopy layer in the wind tunnel, normalized by *H*, the height of the target building model (125 mm).

Under the X-plane view (streamwise direction), regarding the *u* distributions shown in Fig. 16a, the RT building seems to provide a sheltering effect at the leeward of the building. In fact, the *u*_*rms*_ distribution in Fig. 16b shows that turbulent flows are noticeable within those regions.

Also, as shown in Fig. 16a,b, the shape of the plume is directed towards the ground level. Such phenomenon is probably due to the pressure difference that exist within the open field (without building or obstruction) in the downstream region, encouraging flow dissipation. The existence of downstream buildings may have also influenced the travel of plume due to the decrease of wind speed and weak downdraught effect [67].

## Limitation of study

The study considers some limitations in experimental analysis. Therefore, study evaluating the impact of tow direction wind on the wake flow around the two towers. The results reported that the findings have valuable insights into the high-rise buildings effects on the wind flow in their vicinity. Although the findings are exclusive to the actual urban model on which this study was based, the fundamental effects of high-rise structures on wind flows are substantiated through these findings.

The findings of this study are exclusive to the UTM KL campus model, which is representative of the real geometry, and thus subjected to several limitations. Firstly, this study does not compare wake flow with and without the two high-rise buildings, namely RT and MR. Evaluating the impact of wake flows around the two towers compared to a wind field reference containing only low buildings could offer a more comprehensive insight. Secondly, the impact of one high-rise building with a geometric variation of the other one is not considered in this study. Varying the geometric properties or configurations of one high-rise building alone could provide a clearer understanding of the isolated impact each building has on the overall wake dynamics. Third, the analysis does not include the impact of varying the height of a high-rise building on the wake flow. Since height is recognized as a critical factor in the wake flow behaviour, the impact of varying the high-rise building height could provide a more elaborate understanding of the wake flow dynamics in the vicinity of high-rise buildings.

The three limitations addressed previously can be resolved in future experiments and could offer essential insights into how vertical dimensions influence aerodynamic interactions and wake flow generation. The results presented thus far serve as an essential step to understanding wake flow dynamics in the representative urban geometry via wind tunnel experimentation. Therefore, future research incorporates parametrical analysis without controlling the variables that significantly enhance the depth and applicability of findings.

## Conclusion

The parameters *u*, *u*_*rms*_, and SK were used to investigate the strong wind effects on the high-rise buildings through the visualizations of the vertical velocity profiles. The findings showed that the wind patterns are influenced by the configuration or placement of buildings, which correlates with the wind incidences (22.5° and 202.5°). Speed-up winds are spotted at the windward of the building (downdraught effect) and in the gaps between buildings (Venturi effect). Additionally, the strong wind speeds could have been influenced by the design of the buildings’ corner. At RT building’s podium regions experienced strong winds, indicating a need to improve safety measures. These findings can aid in identifying factors that require attention during the planning phase to develop a community–friendly environment. Turbulent wind flow is observed at the leeward of the buildings due to the formation of vortices, whereby the fluctuations in wind are mostly seen. In terms of wake flow, the height of the target building (*H*) had some impact on the distance that vortices traveled. In the MR case, the wakes could be affected up to a minimum distance of *H*, while in the RT case, they could be affected up to a minimum distance of 1.5*H*. Thus, the strong wind effects towards the vicinity of low-rise buildings due to the high-rise building shall not be overlooked.

The present research findings are valuable in providing a practical method to predict both mean and extreme wind speeds that help developers and local councils, especially during the risk assessment stage prior to the urban planning or development, where necessary precautionary steps could be considered.

## Data Availability

The datasets used and/or analyzed during the current study available from the corresponding author on reasonable request.
